# Effect of Butyl Paraben on Oxidative Stress in the Liver of *Mauremys sinensis*

**DOI:** 10.3390/toxics11110915

**Published:** 2023-11-09

**Authors:** Yaru Yin, Zhenzi Xie, Xiao Sun, Xia Wu, Jiliang Zhang, Haitao Shi, Li Ding, Meiling Hong

**Affiliations:** Ministry of Education Key Laboratory for Ecology of Tropical Islands, Key Laboratory of Tropical Animal and Plant Ecology of Hainan Province, College of Life Sciences, Hainan Normal University, Haikou 571158, China; yinyr2023@163.com (Y.Y.); xiezhenzi0630@163.com (Z.X.); sunxiao13732744215@163.com (X.S.); 15185779978@163.com (X.W.); jiliang_zhang@126.com (J.Z.); haitao-shi@263.net (H.S.)

**Keywords:** butyl paraben, turtle, antioxidant enzymes, apoptosis, Nrf2-Keap1 signal pathways

## Abstract

Butyl paraben (BuP) has been widely used as a preservative in the cosmetics, food, and medicine industries. Recently, it has become a new pollutant and has attracted much attention. In order to evaluate the toxic effect of BuP on aquatic animals, Chinese striped-neck turtles (*Mauremys sinensis*) were exposed to BuP solutions with different concentrations of 0, 5, 50, 500, and 5000 µg/L for 20 weeks. The results showed that with an increase in BuP concentration, the activity of antioxidant enzymes (SOD, CAT and GSH-PX) in liver decreased. The expression of key genes in the Nrf2-Keap1 signal pathways first increased and then decreased, while the expression of the HSP70 and HSP90 genes increased. In addition, the liver had an inflammatory reaction. The expression of the BAFF and IL-6 genes increased and then decreased with an increase in BuP concentration, while the expression of P50 and P65 increased significantly. Oxidative stress induced apoptosis, and the expression of pro-apoptosis genes (BAX, cytc, Caspase3 and Caspase9) increased, while the expression of the anti-apoptosis gene Bcl2 decreased. The results provide an important reference for the comprehensive ecological and health risk assessment of environmental BuP.

## 1. Introduction

Parabens include methylparaben (MeP), ethylparaben (EtP), propylparaben (PrP), butylparaben (BuP), benzylparaben (BeP), and other esters of p-hydroxybenzoic acid, which are mostly used as preservatives in food, personal care products, and medicines because of their low reactivity, broad spectrum of antimicrobial activities, high chemical stability (with wide temperature and pH ranges), lack of odor, colorlessness, non-volatility, and low cost of production [1]. Parabens were previously considered safe, until it was reported that these chemicals may be related to breast cancer [2]. Since then, a series of evidence has accumulated from both epidemiologic and experimental studies [3]. In vivo and in vitro studies have shown that parabens have endocrine-disrupting effects and reproductive and developmental toxicity and can affect estrogen activities [4,5,6].

Given the adverse effects of parabens on health, regulations and restrictions have been introduced in many countries to reduce the potential risks caused by parabens. Denmark banned the use of PrP and BuP in children’s cosmetic products in March 2011 [7]. However, according to the European Chemical Substances Information System (2013), 1000–10,000 tons of MeP, 10–1000 tons of EtP and PrP, and about 10 tons of BuP are produced annually. Due to the discharge of industrial wastewater into the environment, organic particles, sludge, sediments, and biological tissues are easily enriched in parabens, which can undergo a biomagnification effect up the food chain [8]. The concentration of parabens in surface water was 3.31–55.2 ng/L in the Yellow River (China) and 15.0–164 ng/L in the Huai River (China), respectively [9], and in fish in Taihu Lake (China), the wet weight of parabens ranged from 532 to 772 pg/g during 2009–2017 [10]. Parabens are predicted to be slightly accumulative in sediment and moderately persistent against sunlight and microbes after being released into the aquatic environment [11]. Recently, parabens have been detected at high levels in humans [12]. The urinary paraben concentrations of samples collected from U.S. and Chinese children were 54.6 and 10.1 ng/mL, respectively [13]. The median lethal concentration values (LC_50_) of paraben at 48 h ranged from 4.0 to 24.6 mg/L in cladoceran (*Daphnia magna*) and from 3.3 to 160.0 mg/L in fathead minnow (*Pimephales promelas*) [14].

Recent ecotoxicological data showing the existence of parabens in the environment have aroused people’s concerns about their biological safety. Some studies have pointed out that a certain concentration of parabens has negative effects on the antioxidant capacity, lipid metabolism, and apoptosis of organisms. When the equilibrium between reactive oxygen species (ROS) production and antioxidant defenses is altered, oxidative stress occurs in organisms, which could lead to lipid peroxidation, DNA damage, and protein degradation in cells [15]. Analyzing oxidative stress markers is important to evaluate the effects of chronic exposure under sublethal doses of parabens. There is a significant positive association between urinary parabens and oxidative stress markers such as malondialdehyde (MDA) or 8-hydroxydeoxyguanosine (8-OHdG) in both maternal and infant urine, suggesting the oxidative stress potentials of parabens [16]. Aquatic organisms are highly sensitive to paraben exposure; in order of sensitivity, the parabens which affect daphnia and fish are BuP > PrP > EtP > MeP [14]. Meanwhile, BuP is easily absorbed and retained in body tissues, and toxicity studies regarding BuP are becoming more frequent. Exposure to BuP through ingestion, inhalation, or skin absorption may damage the endocrine system and induce oxidative stress [17], and it was also reported to cause oxidative stress by inhibiting antioxidants and increasing malondialdehyde (MDA) content in mouse liver [18].

However, the research on BuP is mostly focused on invertebrate, fish, and mammals. Compared to other aquatic animals, turtles are an ideal experimental animal model with a longer life cycle and higher habitat adaptability, which can reflect the environmental quality more truly and comprehensively [19]. Moreover, turtles are advanced animals in the food chain, and they are more sensitive for the assessment of the biomagnification effect of toxins [20]. The Chinese striped-neck turtle (*Mauremys sinensis*) is a kind of freshwater species, found in low-lying, slow-moving ponds, swamps, and streams [21]. The aim of the present study was to evaluate the antioxidant system biomarker responses and oxidative damage present in the liver of *M. sinensis* after 20 weeks of exposure to five different concentrations of BuP (0, 5, 50, 500, and 5000 µg/L). The influence on the oxidant and antioxidant reactions was studied by analyzing the activity of antioxidant enzymes, the content of malondialdehyde (MDA), and the mRNA expression of the main genes involved in the antioxidant pathway. The inflammatory reaction and apoptosis of the liver were also detected. The results provide not only new insights regarding the toxicological mechanism of BuP, but also basic information for the study of ecotoxicology of turtles.

## 2. Materials and Methods

### 2.1. Animals, BuP Exposure, and Sampling

Healthy turtles (1 year old, BW: 98.7 ± 12.8 g) were purchased from Hongwang Agriculture and Culture Company (Haikou, China) and domesticated over 2 weeks. They were cultured in the cement pools (60 cm × 50 cm × 40 cm) at Hainan Normal University and fed on pellet fodder (2% of body weight) every two days. After feeding, the residue was removed, and the water was changed. UVB lights were used during the daytime and the photoperiod was 12L:12D. The ambient pH value was 7.45 ± 0.26, the dissolved oxygen value was 8.40 ± 0.31 mg/L, and the temperature was about 24 °C. After a month of adaptation, the turtles were randomly divided into five groups (0, 5, 50, 500, and 5000 µg/L BuP exposure concentration). BuP (CAS 94-26-8) of analytical grade (≥99% purity) was purchased from Macleans Reagent Company (Shanghai, China). During the experimental period, the water was changed after the turtles were fed and the concentration of BuP was adjusted to the required concentration every day. After 20 weeks of BuP exposure, the turtles were anesthetized via the intraperitoneal administration of 30 mg/kg of body weight of pentobarbital sodium, and then the liver was sampled. Part of the liver was fixed in paraformaldehyde for histological structure, and the rest was quickly frozen in liquid nitrogen and stored in the freezer at −80℃, for biochemical analysis and mRNA expression analysis, respectively. All animal protocols described herein were performed according to the guiding principles of the Hainan Provincial Ecological Environment Education Center Experimental Animal Ethics Committee (No: HNECEE-2019-005).

### 2.2. Biochemical Analysis

About 0.1 g of liver was homogenized with 1 mL of distilled water in an ice bath, then centrifuged for 15 min (4 °C, 4000 rpm). The supernatant was kept for later use. The activities of total superoxide dismutase (T-SOD) (Kit number: A001-3), catalase (CAT) (Kit number: A007-1), glutathione peroxidase (GSH-PX) (Kit number: A005), and MDA (Kit number: A003-1) in the liver were determined using a kit produced by the Nanjing Jiancheng Institute of Bioengineering (Nanjing, China). SOD activity is expressed as the amount of enzyme that inhibits the oxidation of epinephrine by 50 percent, which is equal to 1 U/mg of protein. One unit of CAT activity degrades 1 mmol of H_2_O_2_/min/mg of protein. One indicator of GSH-Px activity is the mmol of NADOH oxidized/min/mg protein. MDA concentrations were determined using 1, 1, 3, 3-tetra ethoxypropane as a standard, and the results were expressed in nmoles of MDA per mg of protein. Protein was estimated according to the method of Read and Northcole [22] using bovine serum albumin as a standard.

### 2.3. Histological Structure

A method was used based on the method described by Ding et al. [23]. Briefly, the fixed livers were dehydrated using gradient ethanol and then transparentized in xylene and embedded in paraffin wax. The hepatic sections (5 μm) were continuously sliced with a slicer (Leica, RM2016, Weztlar, Germany) and then stained with hematoxylin–eosin, sealed with neutral resin, and observed and photographed under an optical microscope (Nikon, Eclipse E100, and DS-U3, Tokyo, Japan).

### 2.4. Total RNA Extraction and qRT-PCR

Total RNA was extracted from the isolated liver using a Trizol^®^ reagent kit (Tiangen biotech Co., Ltd, Beijing, China) according to the standard protocol. Briefly, 100 mg samples were powdered in liquid nitrogen, dissolved completely in 1 mL of TRIzol lysate in a tube, extracted twice with 0.2 mL of chloroform, precipitated with the same volume of isopropanol, and washed twice with 75% ethanol. The precipitation was resuspended in 30 μL of DEPC-treated water. The concentration and integrity of the RNA were measured using a NanoDrop™ One/OneC spectrophotometer (Thermo Scientific, Waltham, MA, USA) and 1.2% agarose gel electrophoresis, separately. cDNA was synthesized from 1 μg of mRNA using the cDNA Synthesis Kit (Takara, Dalian, China), following the manufacturer’s instructions. A quantitative real-time PCR (qRT-PCR) was performed using the Genious 2X SYBR Green Fast qPCR Kit (ABclonal Technology, Wuhan, China) in the LightCycler^®^ 480 Real-Time PCR System (Roche Diagnostics, Basel, Switzerland) with specific primers for the target genes listed in Table 1. All primers were designed based on the gene sequences in our T. s. elegans transcriptome data (accession number: PRJNA94761) using the NCBI website (https://www.ncbi.nlm.nih.gov/tools/primer-blast/, accessed on 4 April 2021) and then synthesized by Sangon Biotech Co., Ltd. (Shanghai, China). β-actin was used as the internal control, and the relative quantification of mRNA expression was calculated using the 2^−ΔΔCt^ method.

### 2.5. Data Analysis

All data were expressed as mean ± S.E. and analyzed in Excel 2020 and SPSS 19.0. After testing the normality and homogeneity, one-way ANOVA was used to analyze the treatment of BuP, and the Duncan method was used to compare the differences between the groups. The data was considered to be significant if the *p* value was less than 0.05.

## 3. Results

### 3.1. Effects of BuP on Antioxidant Enzyme Activity and MDA Content in the Liver

**Figure 1:**
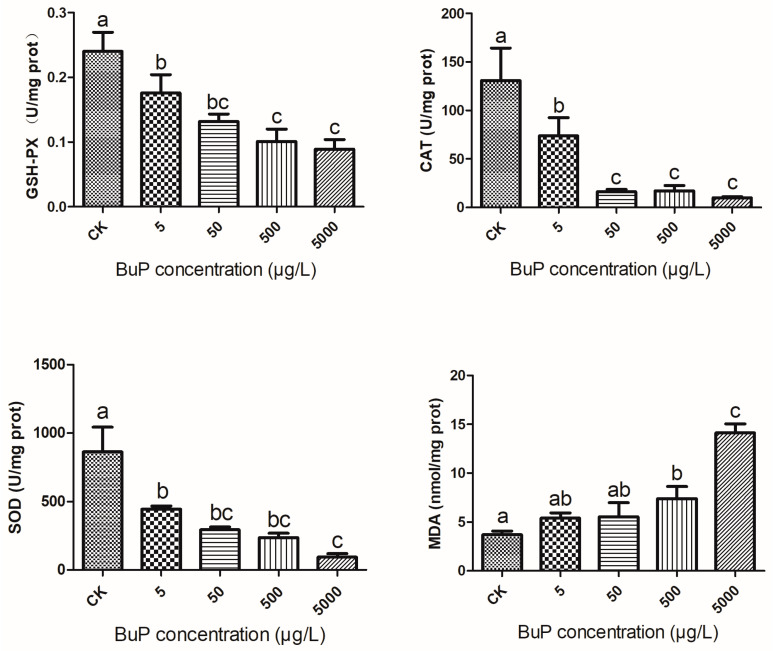
Effect of BuP on the activities of GSH-PX, CAT, and SOD and the content of MDA in the liver of *Mauremys sinensis*. Note: the different letters indicate significant differences between different groups. This is also the case in future figures.

After 20 weeks of stress, the activity of antioxidant enzymes in the liver decreased with the increase in BuP concentration(Figure 1). The activities of GSH-Px, CAT, and SOD in the 5 µg/L BuP group were significantly lower than those of the control (*p* < 0.05). GSH-PX activity in the 500 and 5000 µg/L groups was significantly higher than that of the 5 µg/L group (*p* < 0.05). CAT activity in the 50, 500, and 5000 µg/L groups was significantly higher than in the 5 µg/L group (*p* < 0.05). SOD activity in the 5000 µg/L group was significantly higher than in the 5 µg/L group (*p* < 0.05). As for MDA content, that of the 500 µg/L group was significantly higher than that of the control, though it was significantly lower than that of the 5000 µg/L group (*p* < 0.05).

### 3.2. Effects of BuP on the mRNA Expression of Oxidant Stress Genes in the Liver

**Figure 2:**
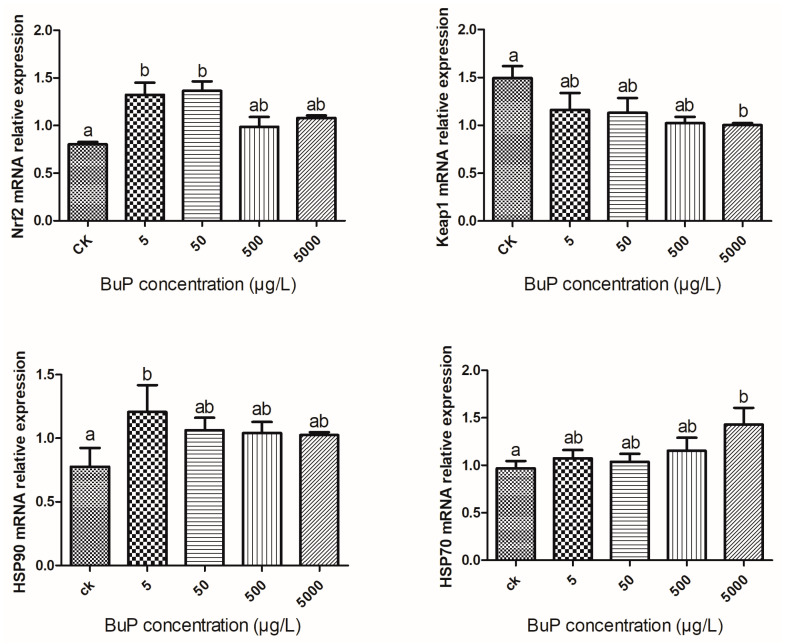
Effect of BuP on relative expression of Nrf2, keap1, HSP90, and HSP70 in the liver of *M. sinensis*.

As shown in Figure 2, after 20 weeks of stress, the relative expression of Nrf2 in the liver in the low-BuP groups (5 and 50 µg/L) was significantly higher than in the control and the high-BuP groups (500 and 5000 µg/L) (*p* < 0.05). The relative expression of Keap1 in the 5000 µg/L group was significantly lower than in the control group (*p* < 0.05).

When the exposure concentration of BuP was 5 µg/L, the relative expression of HSP90 was significantly higher than in the control group (*p* < 0.05). When the concentration of BuP was 5000 µg/L, the relative expression of HSP70 was significantly higher than in the control group (*p* < 0.05).

### 3.3. Effect of BuP on the mRNA Expression of Inflammatory Genes in the Liver

**Figure 3:**
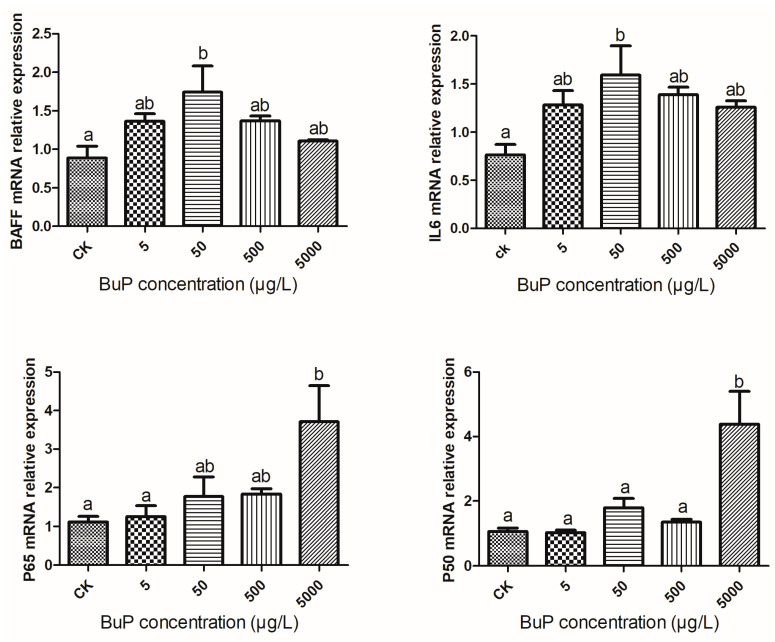
Effect of BuP on the mRNA expression of inflammatory genes in the liver of *M. sinensis*.

As shown in Figure 3, after 20 weeks of stress, the relative expressions of BAFF and IL-6 in the liver were highest in the 50 µg/L group, while the relative expressions of P65 and P50 were highest in the 5000 µg/L group. When the concentration of BuP was 50 µg/L, the relative expressions of BAFF and IL-6 were significantly higher than in the control group (*p* < 0.05), and when the concentration of BuP was 5000 µg/L, the relative expressions of P65 and P50 were significantly higher than in the control group (*p* < 0.05).

### 3.4. Effect of Bup on the mRNA Expression of Apoptosis Genes in the Liver

**Figure 4:**
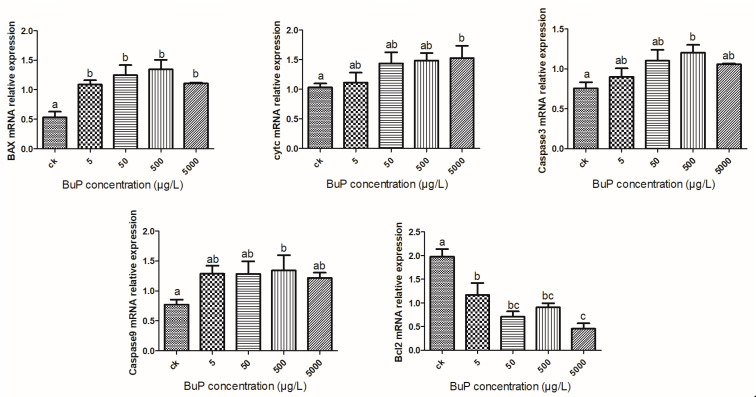
Effect of BuP on the relative expression of apoptosis genes in the liver of *M. sinensis*.

When the concentration of BuP reached 500 µg/L, the relative expression levels of pro-apoptosis genes BAX, Caspase3, and Caspase9 were significantly higher than those in the control group (*p* < 0.05); when the concentration of BuP reached 500 µg/L, the relative expression level of the pro-apoptosis gene cytc was significantly higher than that in the control group (*p* < 0.05), while the relative expression levels of the anti-apoptosis gene Bcl2 were significantly lower than in the control (*p* < 0.05).(Figure 4).

### 3.5. Effect of BuP on the Histological Structure of the Liver

**Figure 5:**
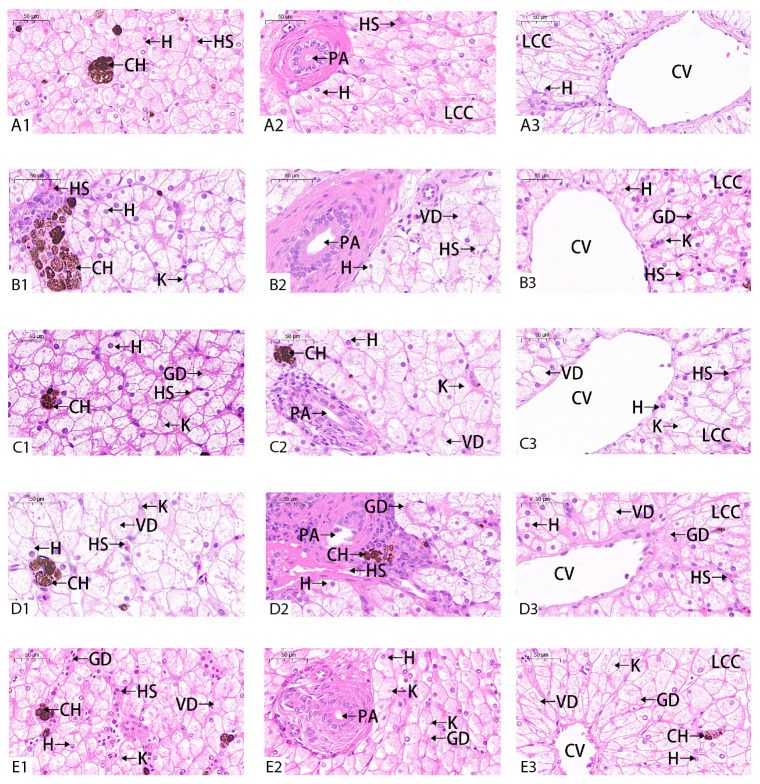
Effect of BuP on the histopathology in the liver of *M. sinensis*. Notes: (**A**) control group; (**B**) 5 g/L BuP group; (**C**) 50 g/L BuP group; (**D**) 500 g/L BuP group; (**E**) 5000 g/L BuP Group. 1, 2,3 mean the different part of the same group. H—hepatocytes; CH—chromatophore; CV—central vein; HS—hepatic sinusoid; PA—portal area; LCC—liver cell cords.

In the control group, the hepatocytes, portal area (PA), and central vein (CV) in the liver were clearly visible and structurally intact, and the liver cell cords (LCC) were arranged neatly (Figure 5A). After 20 weeks BuP stress, the hepatic sinuses in the 5 µg/L group were dilated, the hepatic fibrous tissue in the portal area was obviously proliferated, and liver cell cord was irregularly arranged (Figure 5B). In the 50 µg/L group, the liver cell cord was irregularly arranged, the hepatic sinuses were obviously dilated, and a large number of inflammatory cells were gathered in the portal area (Figure 5C). In the 500 µg/L group, the number of pigment cells were abnormal, the cross-sectional area of central vein was obviously reduced, and there were a large number of inflammatory cells in the portal area. These features were accompanied by liver fibrosis and hepatitis symptoms (Figure 5D). In the 5000 µg/L concentration group, hepatic fibrosis and the inflammatory reaction were also present, and the cross-sectional area of the central vein was significantly decreased (Figure 5E).

## 4. Discussion

A lot of evidence has shown that the physiological indexes related to oxidative stress in aquatic animals can be used as an important basis for monitoring the water quality of pollutants and evaluating the biosafety of pollutants. Numerous xenobiotics could induce oxidative stress where there is insufficient antioxidant activity, leading to the excessive accumulation of free radicals [24]. Since the liver is the prime organ involved in the metabolism of these xenobiotics, it is prone to being attacked by free radicals. The antioxidant defense system is an important self-protection mechanism formed in the long evolutionary process of organisms through the induction of a high expression of phase Ⅱ detoxification enzyme and of hemeoxygenase 1, GSH-PX, SOD, CAT, and other antioxidant proteins. As the primary defense mechanism against free radicals, SOD catalyzes the dismutation of the superoxide anions (O_2_^−^) to O_2_ and H_2_O_2_; CAT plays complementary roles in the elimination of H_2_O_2_; and GSH acts as a primordial electron donor in reducing H_2_O_2_ and organic peroxides, which play an important role in scavenging O_2_^−^ and other reactive oxygen species (ROS) by protecting the organisms from oxidative stress [25]. As the most important product of cell lipid oxidation, reflecting the extent of the damage to the membrane system, MDA can aggravate damage to the cell membrane [26]. After inducing short-term environmental BuP stress (6 d and 12 d) in Nile tilapia (*Oreochromis niloticus*), the activity of SOD in the liver significantly increased, showing that the antioxidant system was activated [27]. Inversely, exposure to BuP for 30 d causes a reduction in non-enzymatic antioxidants and enzymatic antioxidants in the liver of mice, leading to hepatoxicity [18]. Therefore, the dose, the compound being tested, and the duration of exposure influenced the adaptive mechanisms to oxidative stress. In our study, the activities of the antioxidant enzymes (SOD, CAT, and GSH-PX) were negatively correlated with the concentration of BuP (lowest in the 5000 µg/L group); meanwhile, MDA content increased with the concentration of BuP (highest in the 5000 µg/L group), which indicated that the antioxidant system was inhibited and lipid peroxidation was increased. It could be speculated that oxidative stress played an important role in BuP-induced cytotoxicity.

The Nrf2-Keap1 (NF-E2-related factor2 Kelch-like ECH-associated protein1) system is currently considered to be one of the main cellular defense mechanisms against oxidative and alien biological stress, playing a central role in protecting cells from oxidative damage and maintaining the redox balance of cells [28]. This pathway includes not only antioxidant enzyme genes, but also NADPH synthesis [29], the synthesis and regeneration of GSH [30], protease for enhancing recognition, the repair and clearance of damaged proteins, and the inhibition of cytokine-mediated inflammatory factors [31]. Under physiological conditions, Nrf2 binds to cytoplasmic protein partner Keap1 (negative regulatory factor of Nrf2), so that its activity is relatively inhibited. Under the action of oxidative stressors, Nrf2 is decoupled from Keap1 and then transferred into the nucleus to activate antioxidant enzyme genes [28]. Liver function was improved when Keap1 specifically was knocked out in mouse hepatocytes, while the survival rate of hepatocytes was reduced when Nrf2 specifically was knocked out [32]. In this study, the mRNA expression level of Nrf2 increased and then decreased, reaching its peak in the 50 µg/L group, while the relative expression of Keap1 was negatively correlated with the concentration of BuP, which was the lowest in 5000 µg/L group. Therefore, we speculate that the interaction between Nrf2 and Keap1 is indispensable, and activating the expression of antioxidant enzymes may require a congruent change in the expression of the two genes.

Free radical damage is generally combated by inbuilt antioxidant defense systems composed of non-enzymatic compounds, antioxidant enzymes, and heat shock proteins (HSPs), which jointly safeguard the health of the body [33,34]. HSP, as a molecular chaperone, participates in many of the vital physiological processes of cells and organisms, allowing the body to resist environmental pressures [35]. Oxidative stress induces the synthesis of heat shock protein [36], whose levels remain low in a non-stress state and increase rapidly in a stress state [37]. In our study, with an increase in the concentration of BuP, the mRNA expression of HSP70 showed an upward trend, indicating that the turtles try to repair the damage caused by oxidative stress. The same result was observed in *M. sinensis* exposed in ambient ammonia [38]. Meanwhile, HSP90 accounts for 1–2% of the total tissue protein in a normal physiological state [39] and is a highly conserved molecular chaperone that can maintain the spatial conformation of proteins in cells, protect cells, and tolerate stress sources under environmental stress conditions [40]. However, there was no significant difference in the mRNA expression level of HSP90 between the high-BuP group and the control group, presumably because HSP90 is highly conservative and less sensitive to oxidative stress than HSP70.

NF-κB regulates both innate and adaptive immune responses [25]. Considering that antioxidant enzymes play an important role in immune response, we detected the relative expression of key genes (P50, P65, BAFF, and IL-6) in the NF-κB signaling pathway. BAFF is a key regulator in the innate immune system [41], and its activation can lead to the activation of NF-κB, thus increasing the expression of pro-inflammatory cytokines [42]. NF-κB is mainly a dimer composed of p50 and p65 proteins: p50 is the site of binding to DNA, and p65 is involved in the initial regulation of gene transcription, which can promote the binding of p50 to DNA [43]. Previous studies have confirmed that activating a series of pro-inflammatory cytokines (IL-1, IL-6, and IL-12) can enhance the ability of NF-κB to respond to environmental stress [44]. In this study, the relative expression of the BAFF, IL-6, P50, and P65 genes were positively correlated with the concentration of BuP, which showed that the NF-κB signaling pathway was activated, indicating that BuP exposure could induce an inflammatory reaction in *M. sinensis*.

Oxidative stress could induce programmed cell death, such as apoptosis, which regulates cell numbers and removes unwanted and potentially dangerous cells as a defense mechanism [45]. Apoptosis is also regulated by multiple proteins. The B-cell lymphocytic leukemia proto-oncogene (Bcl-2) family is a related protein homologous to Bax, and it is an anti-apoptosis protein that inhibits cell apoptosis [46]. Once the cells receive an apoptotic signal, Bcl2-family proteins will transfer to the mitochondrial membrane from the cytoplasm. On the other hand, the release of pro-apoptotic members of the family, including Bax and cytc, will irreversibly activate downstream caspase to promote the process of apoptosis [47]. When *M. sinensis* was subjected to ammonia, the transcriptional levels of BAX and caspase-3 significantly upregulated, whereas Bcl-2 showed a distinct decrease, and the cleaved caspase-3 was also detected after 24 h of ammonia exposure [48]. In this experiment, with an increase in the concentration of BuP, the mRNA expression level of Bcl-2 in the liver gradually decreased, while the levels of Bax, cytc, caspase-3, and caspase-9 gradually increased, indicating that BuP could induce apoptosis via the caspase-dependent pathway in the hepatocytes of the turtle.

A change in antioxidant enzyme activity and an increase in lipid peroxidation level cause oxidative damage to the body and the appearance of toxicological symptoms [49]. Swelling and the degeneration of hepatocytes occurred, and the hepatic sinuses shrank or even disappeared after ammonia nitrogen stress in *M. sinensis*, which may be related to the decline in antioxidant capacity and cell apoptosis induced by oxidative stress [50]. In our study, the liver showed pathological symptoms such as the swelling of the hepatocytes, the degeneration of pigment cells, the narrowing or even disappearance of central venous vessels, and the abnormal expansion of hepatic sinuses, which indicated that irreversible pathological changes occurred in the liver after BuP stress, and the damage was positively related to the BuP exposure concentration. The results may be related to the inhibition of most enzymes related to antioxidation and the decline of antioxidant capacity [33].

## 5. Conclusions

When the turtles were exposed to BuP, the activities of antioxidant enzymes (SOD, CAT, and GSH-PX) regulated by the Nrf2-Keap1 signal pathway were negatively correlated with the concentration of BuP; these enzyme levels were lowest in 5000 µg/L group and were negatively correlated with the content of MDA in the liver. Meanwhile, as one of the factors affecting the antioxidant system, the relative expression of the HSP70 gene was positively correlated with the concentration of BuP, being highest in 5000 µg/L group. BuP exposure triggered an inflammatory reaction and induced apoptosis, which caused damage to the histological structure of the liver.

## Figures and Tables

**Table 1:** The primers of the main genes that affect oxidant stress.

Target Gene	Forward Primer (5′-3′)	Reverse Primer (5′-3′)
Nrf2	GAGGCCCAGCTTGCACTTA	GGCAGTTTTGAGCAGCCAC
Keap1	GCTCATCGAGTTCGCCTACA	CCTTGACCACGCTGTCGAT
HSP70	CCACCTCTTCGCAGTGTTCT	AAGCCGGGGACAAAAATAGCA
HSP90	TGGAAGGGTTTACCGACGAG	GCCGCTCCTCCTCTTCATAC
P50	GTGACTGCTGGACTGGGAAA	TTTCAGCAAAAGTGCTTGCCA
P65	CTCCAGAAGCCACAGGTTG	GGGGAAGAAGGGGTCAAAGTT
BAFF	CAGAACTAGCAACTCTCCGCAT	ACCCCAGTCCCAGGATAAGAG
IL-6	TAGGTTCTACCGGCTGCACT	CCAGGCATCATGGATCACCA
Bcl-2	GAGGGGATACGATTGGGCTG	AGCAACAGTAGGGGGAGAGA
Bax	GTCGTGGCGCTTTTCTACTT	CCAGCTCGGGGACCTTG
CytC	CTGCGGGCTTCTCTTACACA	TCCATCAGTGTTTCCTCACCC
Caspase-3	TGTGTTAAGTCATGGTGAAGATGGA	AGTTTTGGCTTTCCCACAAGAC
Caspase-9	GACCGAGGATTTGAGGTGGAT	AAAGGGAGTTGCATCGGACT
β-actin	GCACCCTGTGCTGCTTACA	CACAGTGTGGGTGACACCAT

## Data Availability

The data used to support the findings of this study can be made available by the corresponding author upon request.
